# The effects of virtual reality-based interventions on cognitive function, depressive symptoms, and daily functioning in older adults with mild cognitive impairment: a systematic review and meta-analysis of randomized controlled trials

**DOI:** 10.3389/fpubh.2025.1682781

**Published:** 2026-01-16

**Authors:** Liang Chen, Yulin Sun, Qifeng Han, Qian Sun, Zhenping Jiang, Wenbo Ma

**Affiliations:** 1 School of Physical Education and Health, Yili Normal University, Yining, China; 2 School of Physical Education, Yantai University, Yantai, China; 3 Department of Physical Education, Hanyang University, Seoul, Republic of Korea; 4 School of Physical Education, Guangzhou College of Commerce, Guangzhou, China; 5 Department of Sports Science, Hanyang University ERICA, Ansan, Republic of Korea; 6 School of Physical Education, Xi'an University of Architecture and Technology, Xi'an, China

**Keywords:** virtual reality, mild cognitive impairment, exergaming, cognition, depression

## Abstract

**Objective:**

To evaluate the efficacy of virtual reality (VR) interventions in improving cognitive function, depressive symptoms, and daily living ability in older adults with mild cognitive impairment (MCI).

**Methods:**

Eligible randomized controlled trials (RCTs) were systematically retrieved from eight databases and quantitatively synthesized in a meta-analysis. Study quality was appraised using the Cochrane Risk of Bias tool and the GRADE framework.

**Results:**

A total of 24 RCTs involving 1,381 participants were included. VR interventions were associated with moderate improvements in overall cognitive function (SMD = 0.55, 95% CI: 0.36–0.73, *p* < 0.0001), although the certainty of this evidence was rated as low. Subgroup analyses showed that immersive VR and purely cognitive VR were more effective than other types. VR also significantly improved attention (Digit Span Forward: MD = 0.39, *p* = 0.004), processing speed (TMT-A: MD = −4.34, *p* = 0.01), and executive function (TMT-B: MD = −15.76, *p* = 0.009; Digit Span Backward: MD = 0.33, *p* < 0.001). Statistical analysis indicated an absence of significant improvement in daily living performance (SMD = 0.58, *p* = 0.19) and depressive symptoms (SMD = −0.75, *p* = 0.18).

**Conclusion:**

VR interventions, particularly immersive and cognitive-focused types, may enhance cognitive performance in individuals with MCI, especially in attention, processing speed, and executive function. However, current evidence does not support clear benefits for daily functioning or depressive symptoms. Further high-quality studies with long term follow up are needed.

**Systematic review registration:**

https://www.crd.york.ac.uk/PROSPERO/view/ CRD420251002107, ID: CRD420251002107.

## 1 Introduction

At present, dementia affects over 57 million people worldwide, and it is projected that this figure could reach 153 million by 2050, reflecting its rapid escalation into a major global health challenge (1). Mild cognitive impairment (MCI), a condition impacting approximately 15–20% of older adults, represents an intermediate and potentially reversible stage between typical aging and dementia, with an estimated annual conversion rate of 10–15% (2, 3). This stage offers a vital opportunity for timely intervention. Although pharmacological strategies have been investigated, their effectiveness remains modest, with low response rates and insufficient evidence supporting their capacity to decelerate disease progression (4, 5). Consequently, growing attention has been directed toward non-pharmacological strategies such as cognitive training, engagement in physical activity, dietary adjustments, multisensory stimulation, and, more recently, the application of virtual reality (VR)-based interventions (6–8).

With the evolution of digital health tools, VR has gained attention as a potential platform for delivering multidomain interventions. Its immersive and interactive environments allow users to engage in realistic, task-oriented simulations, thereby enhancing motivation and adherence, while also addressing key limitations of traditional methods (such as lack of personalization, difficulty maintaining long-term engagement, and low motivation) (9–11). Although VR-based programs face challenges related to device costs, access barriers, and tolerability issues like cybersickness (9, 10), accumulating evidence suggests that, when implemented appropriately, VR can enhance memory, attention, and executive abilities in older adults with MCI, and may also improve physical performance (12–15). However, significant variability remains in VR formats, including differences in immersion levels, task complexity, and delivery methods. This variability contributes to inconsistent findings and underscores the need for standardized intervention frameworks.

Although several recent systematic reviews and meta-analyses have provided preliminary evidence for the effects of VR interventions in individuals with MCI, their conclusions remain limited in fully characterizing the true magnitude and scope of VR-related benefits. One review included only fully immersive VR and focused primarily on cognitive outcomes (16). Other studies classified VR based solely on device appearance or level of immersion, thereby obscuring the potential additional benefits of physical activity integrated into certain VR formats (17, 18). Moreover, although one review included multiple VR modalities, its primary cognitive outcomes were still inconsistent with those of other similar studies (19). In addition, the small number of included RCTs and the lack of systematic stratification of key dose parameters (intervention duration, weekly frequency, and session length) limited the precision and generalizability of its conclusions. To build upon this evidence base, the present review examines the effectiveness of VR interventions on cognition, functional capacity, and emotional well-being among older adults with MCI. It also analyzes how differences in VR formats, levels of immersion, and implementation strategies may impact intervention efficacy, offering insights for clinical practice, personalized program development, and future policy planning.

## 2 Materials and methods

### 2.1 Protocol and registration

This review adhered to the methodological standards set forth in the Cochrane Handbook for Systematic Reviews of Interventions (20) and was reported in alignment with the PRISMA 2020 statement (21). The protocol was pre-registered with the International Prospective Register of Systematic Reviews (PROSPERO), under registration number CRD420251002107.

### 2.2 Information sources and search

A systematic search was conducted across the following databases: Web of Science, China National Knowledge Infrastructure (CNKI), Ovid (MEDLINE), Cochrane Library, PubMed, Scopus, Research Information Sharing Service (RISS) and the International Clinical Trials Registry Platform (ICTRP). The search included RCTs investigating VR interventions for individuals with MCI, with publications up to January 11, 2025.

The literature search was performed in three steps: (1) systematic searches in each database using Medical Subject Headings (MeSH) and free-text terms; (2) supplementary searches for registered but unpublished trials and manual screening of reference lists from included studies; and (3) all retrieved literature was organized in Endnote X9 for duplicate elimination, followed by screening in accordance with established inclusion criteria.

### 2.3 Eligibility criteria and study selection

Studies were included if they met the following requirements: (1) Participants were adults aged 60 years or above who had been clinically diagnosed with MCI. (2) Intervention: Virtual reality–based programs involving non-pharmacological methods. (3) Control: Groups receiving no intervention, routine care, placement on a waiting list, placebo treatment, or health education. (4) Outcomes: The study assessed at least one indicator related to cognitive function, depressive symptoms, or daily living ability. (5) Duration: The intervention or follow-up phase extended for 4 weeks or longer. (6) Study design: Only RCTs were considered. (7) Exclusion: Articles were excluded if the full text was unavailable, the study was not an RCT, did not focus on MCI participants, used drug-based or non-VR approaches (conventional interventions such as paper-based cognitive training or standard physiotherapy without immersive simulation), or lacked data that could be extracted for analysis.

### 2.4 Data extraction

Two researchers (YS and QS) independently extracted data using a predefined standardized form. A third researcher (LC) reviewed the final dataset to ensure accuracy. Any discrepancies between the two extractors were discussed and resolved by consensus, and when consensus could not be reached, a third reviewer (LC) acted as an arbitrator. Extracted information included study design, participant characteristics, intervention details, and outcome measures. For studies not reporting means and standard deviations directly, data were estimated from medians, ranges, and interquartile ranges using the statistical methods described by Hozo et al. and Wan et al. (22, 23).

Furthermore, guided by the scope of this review and previous literature, VR-based interventions were categorized along two axes: level of immersion and type of functional interaction (12, 18), resulting in two primary subgroups (immersion-based and function-based VR) each comprising three distinct types (Table 1). Control groups were dichotomized into active and passive controls. Active controls involved structured engagement, such as conventional cognitive training, non-VR computer tasks, health education, or physical exercise. Passive controls received no structured therapeutic activities beyond usual care, general nursing, or wait-list placement.

**Table 1:** Classification of VR interventions.

VR category	Operational definition	Examples
Immersion-based VR		
Semi-immersive VR	Systems offering partial immersion using large screens, projections, or motion platforms.	CAVE-like systems, motion-capture screens, panoramic displays.
Fully immersive VR	VR delivered via head-mounted displays that provide 3 60°immersive environments and sensorimotor interaction.	Oculus Rift, HTC Vive, Pico, Meta Quest.
Non-immersive VR	VR presented on conventional screens or televisions, with minimal sensory immersion.	Screen-based exergames, 2D VR tasks.
Function-based VR		
Exergaming VR	VR applications where physical exercise is the primary mode of interaction, and cognitive load is secondary.	Wii Fit, VR cycling, VR balance games requiring full-body movements.
Cognitive-motor VR	VR tasks simultaneously requiring cognitive engagement and purposeful physical movement.	Dual-task stepping, target-reaching with decision-making, coordination tasks.
Purely cognitive VR	VR programs designed exclusively for cognitive training with no meaningful physical movement required.	Memory tasks, attention exercises, visuospatial games performed using controllers or minimal gestures.

### 2.5 Risk and bias

The Cochrane RoB 2 tool was employed to evaluate risk of bias (24). Two reviewers (YS and QS) independently evaluated six domains, including randomization, intervention adherence, outcome data completeness, measurement accuracy, selective reporting, and overall bias. Conflicts in assessment were adjudicated by a third reviewer (LC). Interrater reliability was assessed using Cohen’s kappa. The obtained coefficient (k = 0.88) falls within the 0.81–1.00 range, which according to commonly used benchmarks for kappa (e.g., 0.61–0.80 = substantial, 0.81–1.00 = almost perfect agreement) indicates almost perfect agreement between raters (25).

### 2.6 Statistical analyses

Data synthesis and analysis were carried out in R (v4.4.3). Effect sizes were reported as mean differences (MD) or standardized mean differences (SMD), depending on the type of outcome measure and whether the included studies used comparable but non-identical assessment tools (26). Heterogeneity across studies was assessed using the *I*^2^ statistic, with values of ≤25%, 26–50%, 51–75, and >75% interpreted as low, moderate, substantial, and considerable heterogeneity, respectively (27). Random-effects modeling was utilized in cases where *I*^2^ exceeded 50%, in contrast to fixed-effects modeling, which was applied for comparatively low heterogeneity. For a more comprehensive assessment of heterogeneity, we conducted a series of subgroup analyses for global cognition, stratifying studies by VR functional category (exergaming, cognitive–motor, purely cognitive), immersion level (non-immersive, semi-immersive, fully immersive), type of control group (active vs. passive), intervention duration (≤6, 8–12, >12 weeks), session frequency (≤2 vs. ≥3 sessions per week), and session length (≤40, 41–60, >60 min). We also included study region as a potential influencing factor in a meta-regression to explore whether regional differences may modify the intervention effects. In addition, a sensitivity analysis was conducted by excluding all trials judged to be at high risk of bias, and the pooled effects for the primary outcome and main subgroups were recalculated to evaluate the influence of study quality. To detect potential reporting bias, Egger’s regression asymmetry test was utilized. Furthermore, when 10 or more studies were available for a given outcome, funnel plot symmetry was visually inspected to complement the quantitative assessment (28).

### 2.7 Quality of evidence

The GRADE system was utilized as the basis for appraising the overall quality and reliability of the evidence (29). Only randomized controlled trials were included, so the initial quality rating for each outcome started as high. Two reviewers (YS and QS) independently rated the evidence and resolved any differences through discussion.

## 3 Results

### 3.1 Study selection

**Figure 1:**
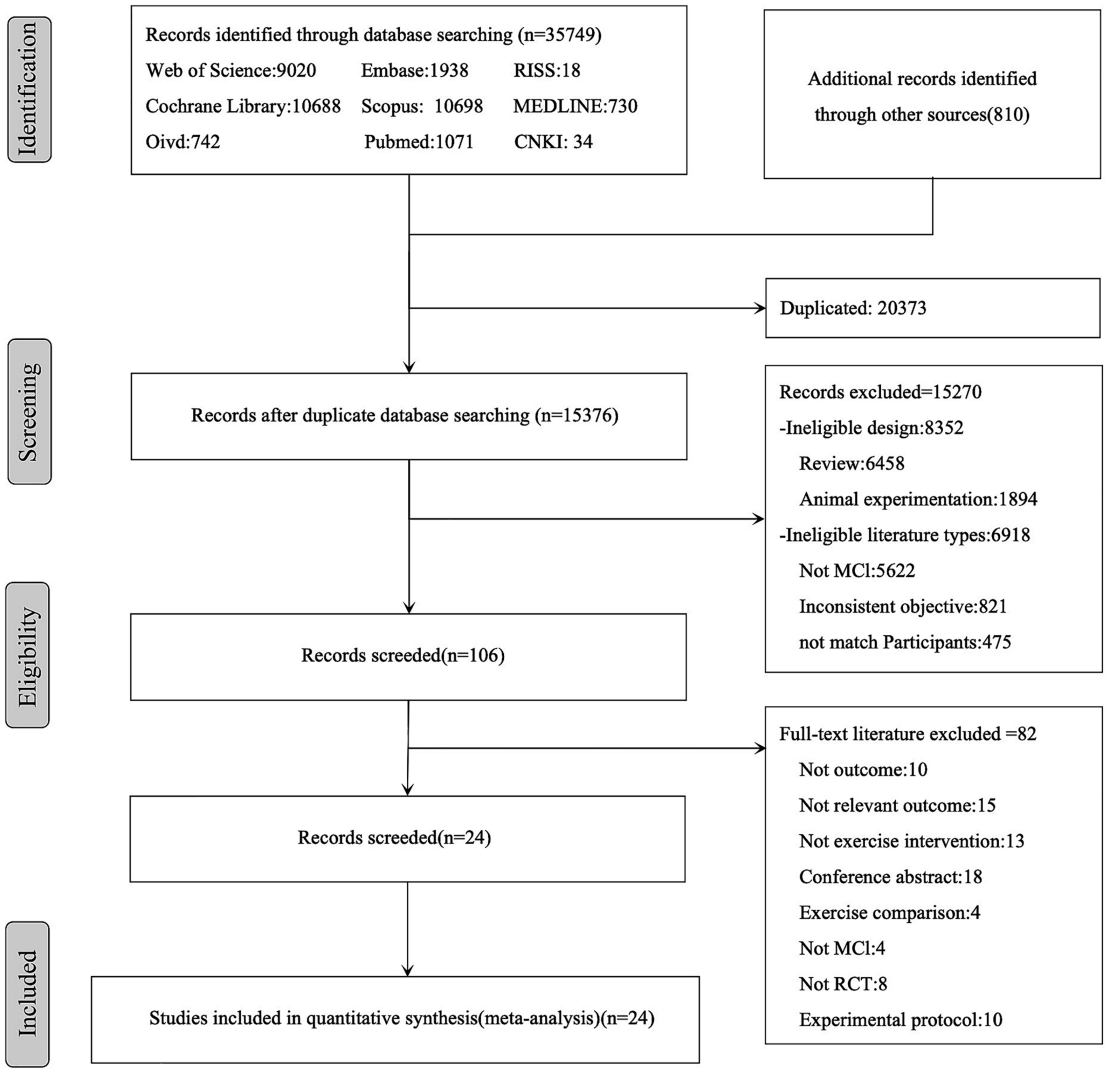
Flow diagram showing the process of study identification and inclusion.

A total of 36,559 records were retrieved through database searches and additional sources. After removing 20,373 duplicates, 15,376 records underwent title and abstract screening, which led to the exclusion of 15,270 non-relevant records. Upon full-text assessment of 106 articles, 82 were found ineligible based on the established criteria. In the end, 24 RCTs were included in the meta-analysis (Figure 1).

### 3.2 Study characteristics

As shown in Table 2, 24 studies with 1,381 participants (mean age: 73.83 years) were conducted between 2014 and 2025. Asia, Europe, and the Americas contributed 15, 6, and 3 studies, respectively (Table 2). Baseline demographic and clinical characteristics were comparable between the VR and control groups across all included trials.

**Table 2:** Classification of VR interventions.

No.	First author (year)	Country	Setting	Sample size (I:C)	Mean age (I:C)	Intervention	Duration (weeks)	Frequency (times)	Session (minute)	Comparison	Outcomes
1	Tarnanas (2014) (30)	Greece	Hospital	32:34	70.5/70.9	Exergaming VR_Immersive VR	20	2	90 min	Cognitive training^a^	MMSE, GDS^1^
2	Hughes (2014) (31)	United States	Community	10:10	78.5/76.2	Exergaming VR_Semi-Immersive VR	24	1	90 min	Health education^a^	CAMCI, IADL
3	Djabelkhir (2017) (40)	France	Community	9:10	78.2/75.2	Purely Cognitive VR_Non-Immersive VR	12	1	90 min	Computerized cognitive engagement^a^	MMSE, GDS^2^, TMT-A
4	Savulich (2017) (41)	United Kingdom	Memory clinics	21:21	75.2/76.9	Purely Cognitive VR_Non-Immersive VR	4	2	60 min	Normal activities^b^	MMSE
5	Delbroek (2017) (32)	Belgium	Care center	10:10	86.9/87.5	Exergaming VR-Semi-Immersive VR	6	2	18-30 min	Usual care^b^	MOCA, TUG
6	Park (2017) (15)	Korea	Community welfare center	39:39	65.9/67.9	Exergaming VR_Non-Immersive VR	10	3	30 min	CCT CoTras Program^a^	DSF, DSB, TMT-B
7	Choi (2019) (34)	Korea	Welfare center	30:30	77.2/75.3	Exergaming VR_Immersive VR	6	2	60 min	Health education^a^	MOCA, TUG
8	Liao et al. (2019) (49)	China	Communities	18:16	72.2/70.7	Cognitive-motor VR _Immersive VR	12	3	60 min	Sport + cognition^a^	TMT-A, TMT-B
9	Schwenk et al. (2016) (35)	United States	Memory disorders clinic	11:9	79.0/77.8	Exergaming VR_Semi-Immersive VR	4	2	45 min	Normal activities^b^	MOCA
10	Thapa et al. (2020) (36)	Korea	Care center	34:34	72.6 /72.7	Exergaming VR_Immersive VR	8	3	100 min	Health education^a^	MMSE, TUG, TMT-A, TMT-B
11	Park et al. (2020) (42)	Korea	Community	18:17	75.8/77.2	Cognitive-motor VR_Immersive VR	6	5	30 min	Cognitive rehabilitation^a^	MOCA, DSF, DSB, TMT-A, TMT-B
12	Liao et al. (2020) (50)	China	Communities	18:16	75.5/73.1	Cognitive-motor VR_Immersive VR	12	3	60 min	Sport + cognition^a^	MOCA, IADL
13	Park et al. (2020) (43)	Korea	Memory disorders clinic	10:11	71.8/69.4	Purely cognitive VR_Immersive VR	12	2	30 min	Normal activities	MMSE, SGDS-K, DSF, DSB
14	Torpil et al. (2021) (44)	Turkey	Occupational therapy department	30:31	70.1/70.3	Purely cognitive VR_Immersive VR	12	2	45 min	Computer training^a^	LOTCA-G
15	Liu et al. (2022) (37)	China	Community	16:17	74.6/73.4	Exergaming VR_Semi-Immersive VR	12	3	50 min	Usual care^b^	MOCA, TMT-A, TMT-B
16	Yang et al. (2022) (13)	Korea	Regional cohorts	33:33	72.5/67.9	Purely cognitive VR_Immersive VR	8	3	100 min	Health education^a^	MMSE, TMT-A
17	Arshad et al. (2023) (38)	Pakistan	Hospital	26:25	62.8/63.2	Exergaming VR_Immersive VR	6	5	30 min	Stretching^a^	MOCA, TMT-A, TMT-B
18	Hwang et al. (2023) (45)	China	Hospital	46:56	65 above	Purely cognitive VR_Non-Immersive VR	24	1	50 min	Health education^a^	MDRS, ADLs, TUG
19	Buele et al. (2024) (51)	Ecuador	Care center	17:17	75.4:77.3	Cognitive-motor VR_Immersive VR	6	2	40 min	Sport + cognition^a^	MOCA, SGDS, IADL
20	Kwan et al. (2024) (52)	China	Community	146:147	75.2:73.9	Cognitive-motor VR_Immersive VR	8	2	60 min	General nursing^b^	MOCA, DSF, DSB, TUG
21	Park (2025) (46)	Korea	Senior centers	30:30	69.0:70.5	Purely cognitive VR_Immersive VR	4	2	15 min	Wait-list^b^	DSB, TMT-B
22	Graessel et al. (2024) (47)	Germany	Hospital	44:45	73.4:73.5	Purely cognitive VR_Non-Immersive VR	24	3	40 min	Computer training^a^	MOCA
23	Ip et al. (2025) (39)	United Kingdom	Community	9:9	73.6:80.0	Exergaming VR_Immersive VR	8	2	45 min	Exercise^a^	HK-MoCA, TMT-A, TMT-B, TUG
24	Zheng et al. (2025) (48)	China	Nursing home	33:33	80.8:79.5	Purely cognitive VR_Immersive VR	12	2	45 min	Usual care^b^	MMSE, GDS, IADL

I, intervention group; C, control group; a, active control; b, passive control; ADLs, activities of daily living; CAMCI, The Computerized Assessment of Mild Cognitive Impairment; DSF, digit span forward; DSB, digit span backward; GDS1, Geriatric Depression Scale; GDS2, Goldberg Depression Scales; HK-MoCA, Hong Kong Montreal Cognitive Assessment; IADL, The Instrumental Activities of Daily Living; K-MMSE, Korean version of Mini-Mental State Examination; LOTCA-G, Loewenstein Occupational Therapy Cognitive Assessment-Geriatric; MDRS, The 36-item Mattis Dementia Rating Scale; MMSE, Mini-Mental State Examination; MoCA, Montreal Cognitive Assessment; SGDS, Spanish version of the Short Form of Geriatric Depression Scale; SGDS-K, Korean version of the Geriatric Depression Scale-Short form; TMT-A, trail making test part A; TMT-B, trail making test part B; TUG, timed up and go.

VR-based interventions were categorized into three main types: exergaming VR (*n* = 10) (30–40), purely cognitive VR (*n* = 10) (13, 41–49), and cognitive-motor VR (*n* = 4) (50–53). Among these, immersive VR systems were most frequently employed, particularly in exergaming and cognitive-motor interventions, while non-immersive VR platforms were more commonly used in cognitive-only interventions. Semi-immersive systems were occasionally utilized in both the exergaming and cognitive-motor categories.

Regarding intervention duration, 13 studies lasted 12 weeks or less, 8 studies ranged from 13 to 24 weeks, and 3 studies exceeded 24 weeks. Sessions were held between one and five times per week, with durations varying from approximately 15 min to 100 min.

### 3.3 Risk of bias within studies

**Figure 2:**
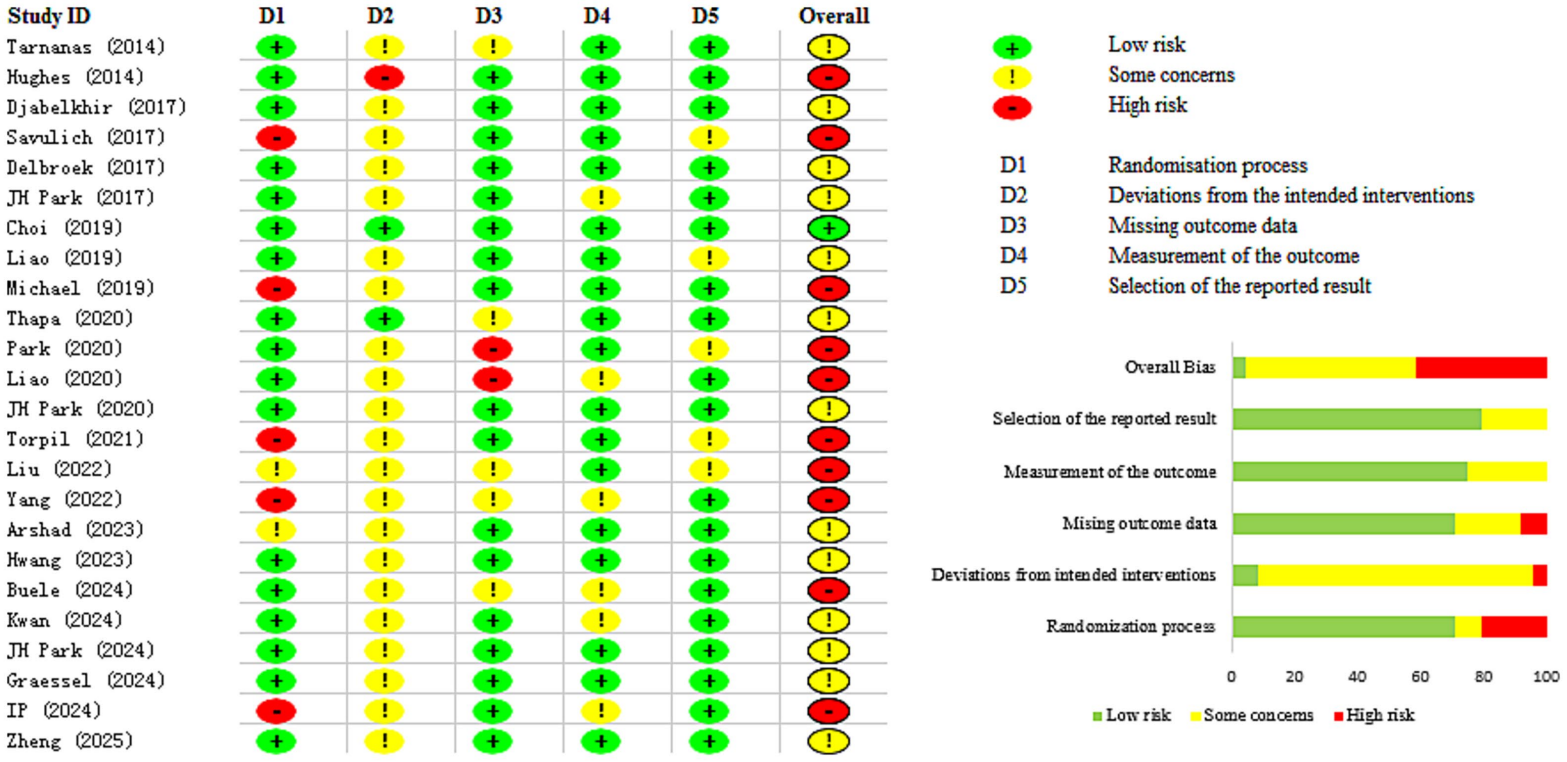
Risk of bias assessment.

Among the studies included in the analysis, one was judged to exhibit a low risk of bias (34), whereas 10 were assessed as high risk (13, 31, 35, 38, 40, 42, 43, 45, 51, 52). The remaining 13 studies were flagged as having some methodological concerns (30, 32, 33, 36, 39, 41, 44, 46–50, 53). A study was categorized as high risk if it presented at least one domain with a high-risk rating or showed concerns in three or more domains (Figure 2).

### 3.4 Synthesis of results

#### 3.4.1 Primary outcomes

##### 3.4.1.1 Cognitive function

**Figure 3:**
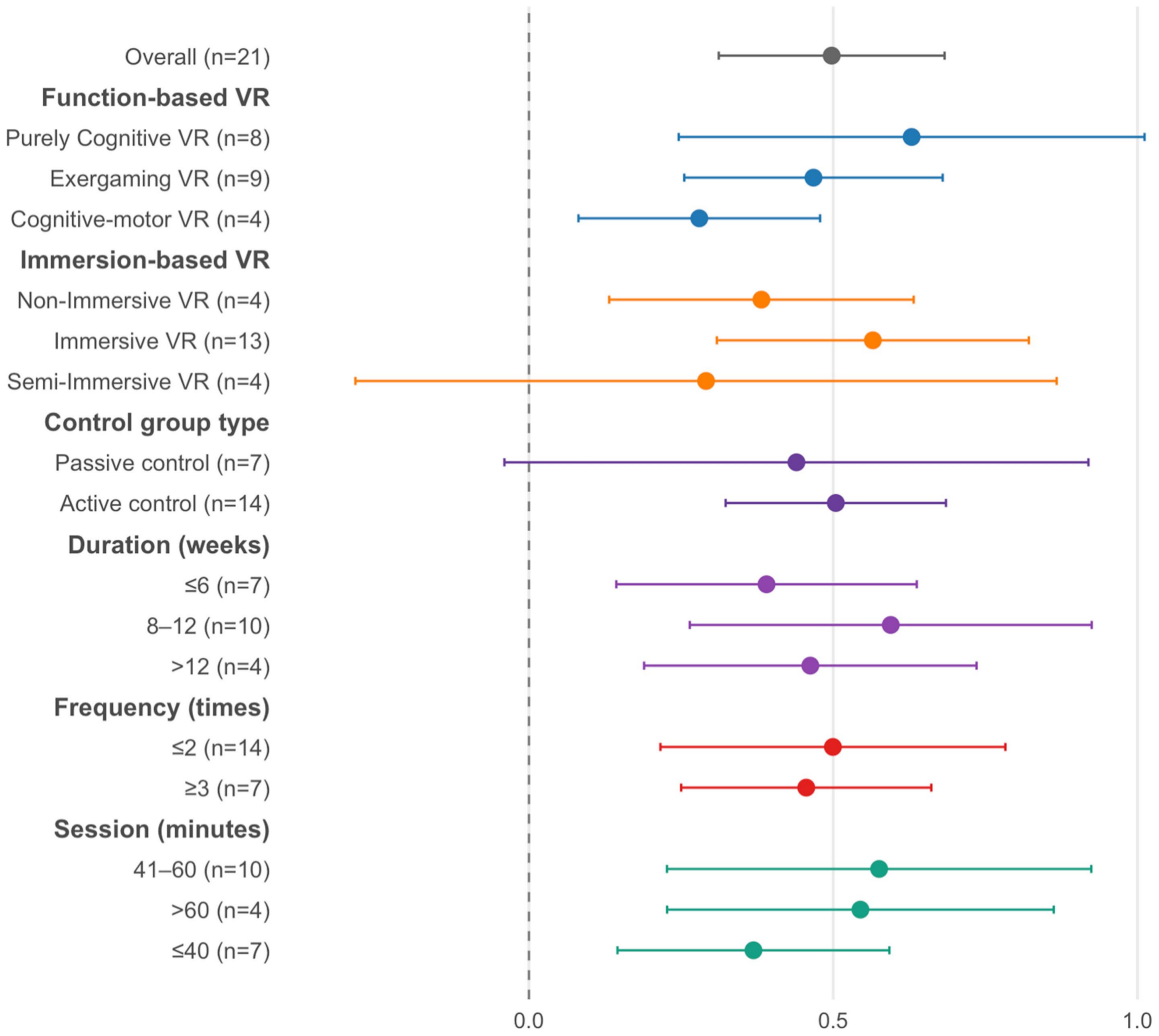
The effect of VR on cognition.

A total of 21 studies assessed cognitive outcomes associated with VR-based interventions (Figure 3). Given the substantial heterogeneity among the included studies (*I*^2^ = 54%, *p* = 0.0016), a random-effects approach was utilized. Meta-analysis results indicated a moderate improvement in cognitive performance for participants receiving VR interventions compared with controls (SMD = 0.55, 95% CI: 0.36–0.73, *p* < 0.0001). Sensitivity analysis excluding the 10 high-risk trials (leaving 11 trials) yielded a slightly larger pooled effect (SMD = 0.69, 95% CI: 0.43–0.95; *I*^2^ = 52.8%). This consistency supports the robustness of the primary findings, indicating that the observed benefits were not driven by low-quality evidence.

To further explore potential sources of heterogeneity and to characterize the conditions under which VR may be most effective, we conducted a series of prespecified subgroup analyses. Subgroup analysis revealed that both purely cognitive VR (SMD = 0.63, 95% CI: 0.25–1.01, *p* = 0.001) and immersive VR (SMD = 0.57, 95% CI: 0.31–0.82, *p* = 0.0001) produced significantly greater effects than other intervention formats. When stratified by control type, VR showed significant benefits over active controls (SMD = 0.50, 95% CI: 0.32–0.69, *p* < 0.001). Unexpectedly, the comparison with passive controls did not initially reach significance (SMD = 0.44, 95% CI: −0.04–0.92, *p* = 0.073). However, sensitivity analysis excluding high-risk trials revealed a robust and larger effect against passive controls (SMD = 0.84, 95% CI: 0.30–1.37, *p* = 0.002), suggesting that low-quality studies may have obscured the true benefit in this subgroup. Interventions lasting between 8 and 12 weeks (SMD = 0.59, 95% CI: 0.26–0.92, *p* = 0.0004), delivered no more than twice weekly (SMD = 0.50, 95% CI: 0.22–0.78, *p* = 0.0005), and with session durations of 41–60 min (SMD = 0.58, 95% CI: 0.23–0.92, *p* = 0.001) were also associated with superior efficacy compared with other subgroups.

In addition, to further explore potential sources of heterogeneity, we conducted a meta-regression with study region as a moderator. Study region explained about 17% of the between-study heterogeneity (*R*^2^ = 17.0%), with trials conducted in Europe showing larger effect sizes than those from America (*β* = 0.66, 95% CI 0.02–1.31, *p* = 0.044), whereas the difference between studies from Asia and America was not statistically significant (*β* = 0.46, 95% CI − 0.15–1.07, *p* = 0.14). Nevertheless, residual heterogeneity remained moderate (residual *I*^2^ = 49.5%), suggesting that geographic factors only partially accounted for the observed variability.

**Figure 4:**
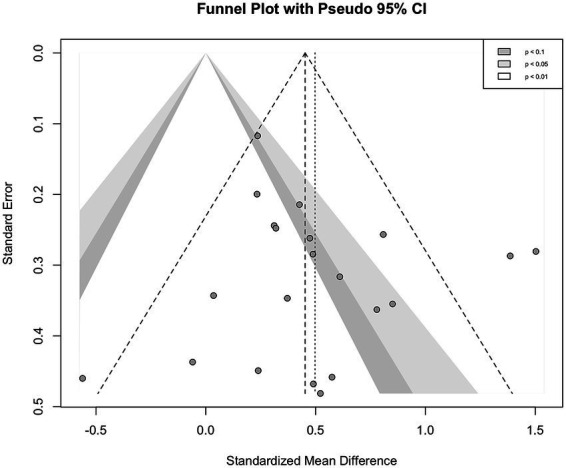
Funnel plot on cognition.

The funnel plot displayed an overall symmetrical pattern, suggesting an absence of notable asymmetry (Figure 4). In addition, Egger’s regression test revealed no evidence of significant publication bias (*t* = 0.83, *p* = 0.41).

Forest plot showing the primary meta-analysis of global cognitive outcomes, including all eligible studies.

#### 3.4.2 Secondary outcomes

**Figure 5:**
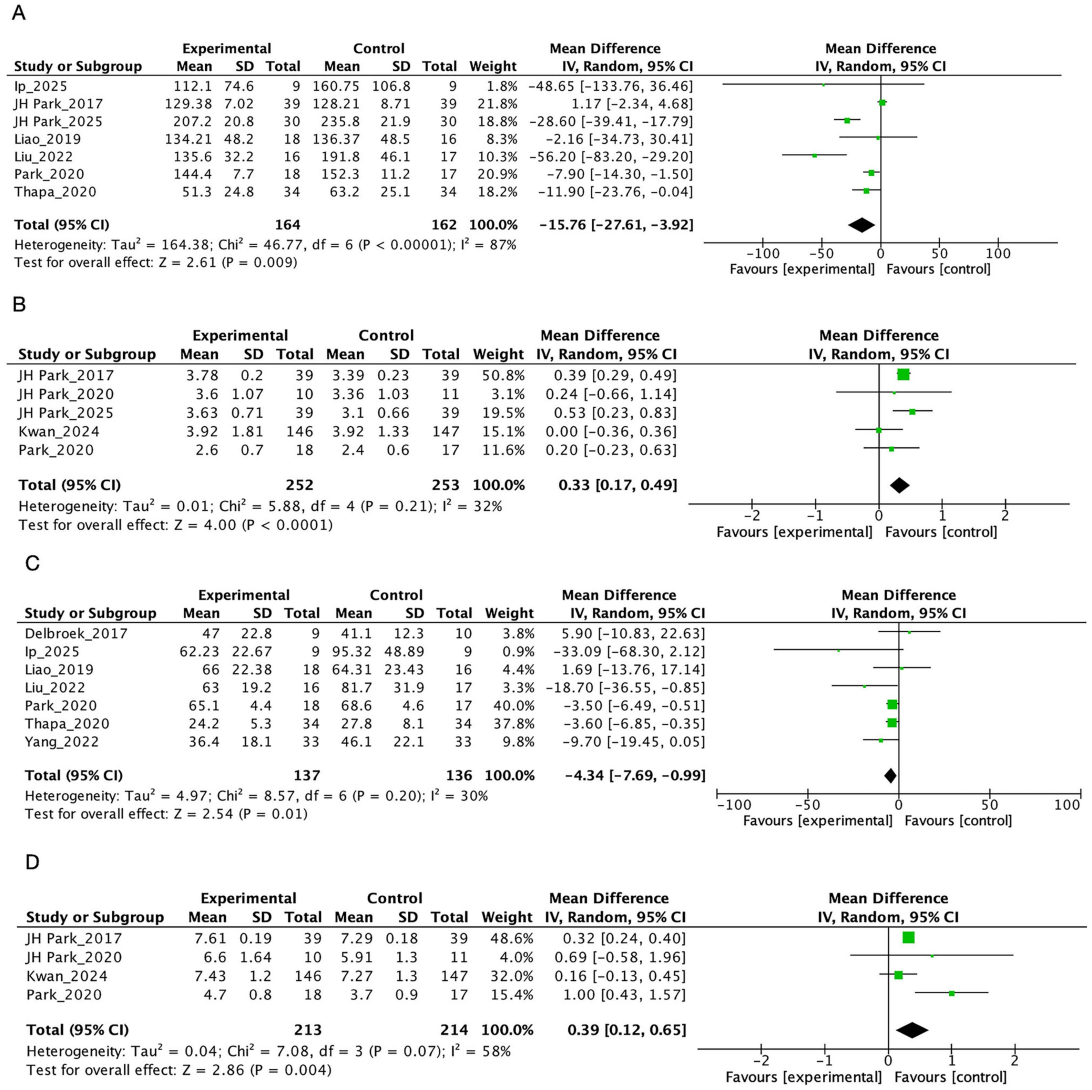
The effect of VR on other cognition area.

In terms of executive function, VR interventions showed significant improvements in scores on the TMT-B (MD = −15.76, 95% CI: −27.61 to −3.92, *p* = 0.009) (Figure 5A) and the DSB (MD = 0.33, 95% CI: 0.17 to 0.49, *p* < 0.001) (Figure 5B). However, the TUG (MD = −0.84, 95% CI: −2.50 to 0.83, *p* = 0.32) did not show significant effects.

Additionally, positive effects were observed in attention and processing speed. The TMT-A (MD = −4.34, 95% CI: −7.69 to −0.99, *p* = 0.01) (Figure 5C) showed significant improvement, and the DSF (MD = 0.39, 95% CI: 0.12 to 0.65, *p* = 0.004) (Figure 5D) also demonstrated significant improvement.

**Figure 6:**
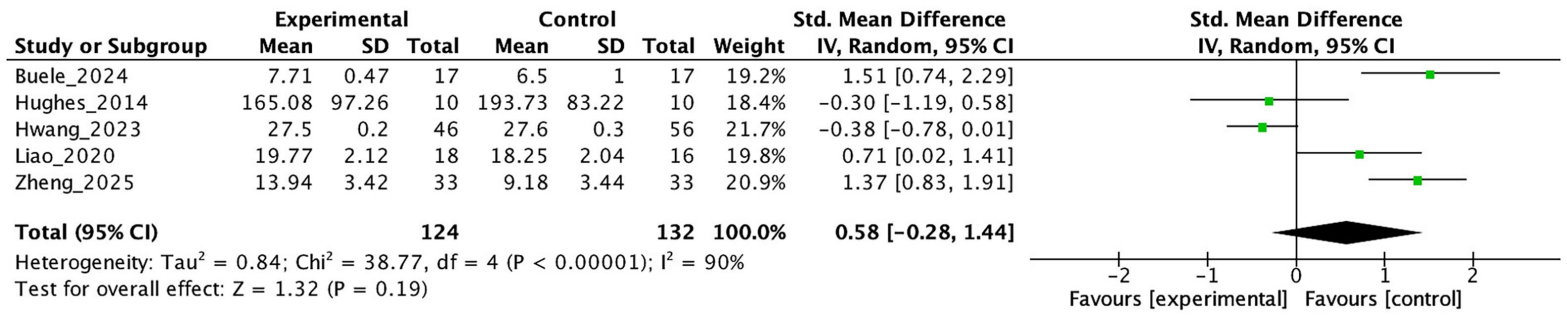
The effect of VR on daily functioning.

**Figure 7:**
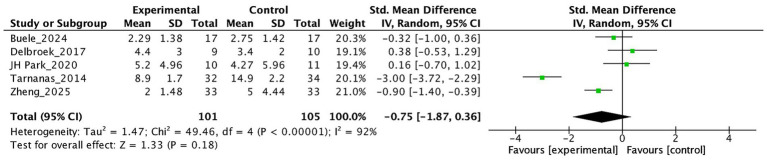
The effect of VR on Depression.

Figures 6, 7 present the effects of virtual reality interventions on functional ability and depressive symptoms among individuals with MCI. No statistically significant improvement was observed in daily functioning (SMD = 0.58, 95% CI: −0.28 to 1.44, *p* = 0.19) or depressive symptoms (SMD = −0.75, 95% CI: −1.87 to 0.36, *p* = 0.18) when compared to control conditions.

### 3.5 Quality of evidence within studies

GRADE analysis suggested that the level of certainty for evidence on the effects of VR interventions on cognition, attention, and processing speed among MCI participants was low (Table 3). Despite including 21 studies on cognitive outcomes, the certainty of evidence was considered low due to notable heterogeneity (*I*^2^ > 50%) and significant bias risks in several trials, especially those with poor attrition management and lack of allocation concealment. Regarding executive function, evidence from TMT-B was downgraded to very low due to substantial heterogeneity and the involvement of over three high-risk studies. In contrast, the DSB related evidence was considered moderate since fewer studies were flagged for high bias risk.

**Table 3:** Research quality grade assessment results.

Certainty assessment							No. of patients		Effect		Certainty	Importance
No. of studies	Study design	Risk of bias	Inconsistency	Indirectness	Imprecision	Other considerations	Virtual reality	Control	Relative (95% CI)	Absolute (95% CI)		
Global cognition												
21	Randomized trials	Serious^a^	Serious^b^	Not serious	Not serious	None	581	617	-	SMD 0.55 SD higher (0.37 higher to 9.76 higher)	⨁⨁◯◯ Low^a^^,^^b^	Important
TMT-A												
6	Randomized trials	Very serious^c^	Not serious	Not serious	Not serious	None	121	119	-	SMD 0.4 SD lower (0.7 lower to 0.1 lower)	⨁⨁◯◯ Low^c^	Important
Digit span forward												
4	Randomized trials	Serious^d^	Serious^b^	Not serious	Not serious	None	213	214	-	SMD 1.27 SD higher (0.82 higher to 1.72 higher)	⨁⨁◯◯ Low^b^^,^^d^	Important
TMT-B												
7	Randomized trials	Very serious^c^	Serious^b^	Not serious	Not serious	None			-	MD 15.76 lower (27.61 lower to 3.92 lower)	⨁◯◯◯ Very low^b^^,^^c^	Important
Digit span backward												
5	Randomized trials	Serious^e^	Not serious	Not serious	Not serious	None			-	MD 0.33 higher (0.17 higher to 0.49 higher)	⨁⨁⨁◯ Moderate^e^	Important

CI, confidence interval; MD, mean difference; SMD, standardized mean difference.

a absence of an appropriate analytical method to address attrition during the intervention, and lack of allocation concealment.

b *I*^2^ > 50%, indicates substantial heterogeneity and suggests considerable inconsistency among the study results.

c Three studies exhibited a high risk of bias.

d One study exhibited a high risk of bias, and the risk of bias in two studies was concerning.

e One study had a high risk of bias, while the risk of bias in the remaining studies was concerning.

For processing speed, the certainty of evidence remained low, mainly due to methodological flaws such as inadequate randomization concealment and moderate heterogeneity. Similarly, the evidence supporting attention outcomes was judged to be of low quality because three studies showed a high risk of bias.

## 4 Discussion

In this meta-analysis, we evaluated how VR-based interventions influence cognitive performance, daily functioning, and depressive symptoms among individuals with MCI. Our findings demonstrate that VR interventions yield moderate improvements in global cognitive function compared with controls. Notably, this cognitive benefit remained robust even when VR was compared with active control groups. However, given the low certainty of evidence assessed by GRADE, these cognitive benefits warrant cautious interpretation. Conversely, current evidence does not support significant benefits for daily functioning or depressive symptoms.

In particular, the improvement in cognitive function aligns with previous meta-analyses (17, 18) but contrasts with findings from another study (19). Subgroup analyses further revealed that purely cognitive VR interventions and immersive VR approaches yielded greater cognitive benefits than exergaming VR or semi-immersive VR. Purely cognitive VR interventions generally comprise structured modules designed to enhance specific abilities—such as memory, attentional flexibility, and executive control—targeting the primary cognitive deficits found in individuals with MCI (41, 48). These interventions are often systematic and tailored, allowing for targeted stimulation of neurocognitive circuits. In contrast, exergaming VR—though effective in promoting physical activity—provides indirect and fragmented cognitive stimulation, which may be insufficient for systematically engaging higher-order brain regions (54, 55). Immersive VR, by leveraging head-mounted displays and 3D virtual environments, creates highly interactive experiences that foster task engagement and contextual memory encoding. This immersive quality may enhance neuroplasticity (34, 39). Research indicates that immersive, context-specific learning in VR settings promotes functional reorganization within critical cognitive regions, including the hippocampus and prefrontal cortex, which is a particularly important mechanism in the rehabilitation of individuals with MCI (56). Moreover, combining purely cognitive and immersive elements activates multiple sensory pathways (e.g., visual, auditory, motor), thereby promoting multisensory integration and improving the coordination of perception-cognition-action systems (44, 47). In contrast, semi-immersive VR and non-immersive VR (such as those using large screens or standard computers) lack 3D spatial experiences and provide limited sensory stimuli, making it difficult to engage higher-level cognitive processing.

Regarding intervention duration, interventions lasting 8–12 weeks, with a frequency of up to 2 sessions per week and session durations of 41–60 min, were found to have the most significant effects. Among these, the most common intervention design was 2 sessions per week, each lasting 60 min. Cognitive improvement depends on neuroplasticity, which involves the restructuring and functional enhancement of synaptic connections between neurons (57). Studies suggest that the brain can gradually establish new cognitive pathways and consolidate cognitive gains through regular training lasting 8–12 weeks (58–60). A duration shorter than 6 weeks may not lead to stable neural changes, while interventions longer than 12 weeks may diminish the effects due to decreased motivation and accumulated fatigue (61). Moreover, for MCI patients, sufficient recovery time after cognitive training is essential to allow the brain to integrate and stabilize new information. Intervening twice per week prevents over-stimulation of the central nervous system, reduces psychological fatigue, physical burden, and enables the training to follow the “activation-recovery-re-activation” cycle, which aligns with the theory of cognitive consolidation. A higher frequency (more than four times per week) may lead to physical and mental fatigue or reduced adherence. Additionally, several studies have identified 40–60 min as the optimal duration for cognitive training, as this period is ideal for neural activation and sustained attention (13, 49). Sessions shorter than 40 min may be insufficient for stimulation, while those exceeding 60 min may cause cognitive overload, reduced attention, and accumulated fatigue, especially among MCI patients.

It is also worth noting that the characteristics of VR interventions necessitate that single-session durations not be too long. Although VR interventions are highly interactive, they can induce “VR fatigue” or “motion sickness,” particularly during prolonged or frequent exposure. Therefore, moderate frequency and duration help balance intervention intensity and comfort, improving acceptability and sustainability. Thus, the most commonly used intervention design in practice is 8–12 weeks, with 2 sessions per week, each lasting 60 min. This combination offers high feasibility and real-world adherence, and aligns with international clinical intervention guidelines, such as those from the WHO and ACSM.

Methodological limitations present in high-risk studies, such as insufficient allocation concealment and inadequate handling of participant attrition, could theoretically inflate the estimated intervention effects. However, contrary to this general expectation, sensitivity analyses excluding high-risk trials revealed an increased effect size, with the SMD rising from 0.55 to 0.69. This strengthening of efficacy suggests that the relatively rigorous protocols and potentially superior adherence in the retained low-risk trials may have unmasked the true therapeutic potential of VR, whereas methodological flaws in high-risk studies likely obscured these benefits.

This study examined the effects of VR interventions on executive function, attention, processing speed, daily living ability, and depressive symptoms. The results showed that VR significantly improved certain aspects of executive function, as well as attention and processing speed. However, no significant effects were observed on TUG, daily functioning, or depressive symptoms.

Specifically, VR interventions significantly enhanced MCI patients’ performance on the Digit Span Forward and TMT-A, which is consistent with previous meta-analyses (20). In contrast, other measures of executive function, such as the TUG test, did not show significant improvement. These findings suggest that VR interventions may be more effective in improving lower-level cognitive processes (e.g., attention and processing speed), while their effects on more complex executive tasks may be limited. For example, Yang (2022) also found significant post-intervention improvements in the VR group on the TMT-A (19). Executive function represents a higher-order cognitive domain involving working memory, cognitive flexibility, and inhibitory control (61). Because VR-based interventions often rely on task-driven interactive experiences, they may be particularly well-suited for enhancing foundational cognitive functions. However, improving higher-level executive function may require more targeted content or longer-term interventions. Furthermore, the limited effects on daily living ability may be due to the fact that IADL tasks demand not only cognitive coordination but also sufficient physical capability—areas that may not be fully addressed by VR alone.

Additionally, VR interventions showed limited effects on improving IADL of MCI patients. Although VR interventions can promote physical activity and improve some physical functions, relying solely on VR interventions may not significantly enhance MCI patients’ independence in daily living, particularly in more complex physical functions such as balance and muscle strength. Therefore, combining VR interventions with physical function training, particularly in areas such as balance and strength training, may achieve superior outcomes in strengthening daily activity performance and autonomy in MCI populations.

Regarding depression symptoms, VR interventions did not show significant effects in reducing depression in MCI patients. While VR interventions provide an interactive and immersive experience, their impact on mood and psychological health may require longer intervention periods or integration with other psychological interventions. The improvement of depression symptoms is often a complex process, which may require a combination of VR-based cognitive behavioral therapy and social activities, among other strategies, to achieve more significant effects (62).

This review has several limitations. First, the majority of included studies were conducted in Asian countries, with limited representation from Western regions, which may restrict the generalizability of findings to broader global populations. Second, although publication bias tests were negative, their power is reduced in the presence of high heterogeneity (*I*^2^ > 50%). Furthermore, the multiplicity of subgroup analyses warrants caution regarding potential Type I errors. Third, the inconsistent reporting of adverse events precludes a definitive safety assessment. Finally, the predominance of short-term follow-ups limits our understanding of the durability of effects, and broader neuropsychiatric outcomes (e.g., anxiety, sleep) remain under-investigated. Consequently, our conclusions primarily reflect short-term efficacy. Future trials should prioritize multi-center designs with extended follow-ups to determine the longevity of cognitive gains. Safety protocols must be standardized, systematically monitoring cybersickness and fatigue. Moreover, expanding outcome measures to include anxiety, quality of life, and real-world functional endpoints will be crucial for establishing the clinical value of VR in MCI care.

## 5 Conclusion

This review assessed VR-based interventions for people with MCI across multiple domains, including global cognition, executive abilities, attention, processing speed, activities of daily living, and depressive symptoms. Current evidence of low certainty suggests that VR confers moderate benefits on global cognitive outcomes, particularly when delivered through purely cognitive and immersive formats. Our analyses further identified a potential optimal therapeutic window: interventions lasting 8–12 weeks, with a frequency of twice weekly and session durations of 41–60 min, appear most effective. In contrast, no significant improvements were observed for daily functioning or depressive symptoms. To advance the field, future research should prioritize larger samples with extended follow-up to evaluate the durability of effects. Furthermore, expanding outcome targets to include broader neuropsychiatric measures and real-world functional endpoints is essential. Such efforts will strengthen the evidence base, enhance clinical applicability, and inform the standardization of VR protocols for integration into routine MCI care.

## Data Availability

The original contributions presented in the study are included in the article/Supplementary material, further inquiries can be directed to the corresponding authors.
