# Supplementary material for: The effects of virtual reality-based interventions on cognitive function, depressive symptoms, and daily functioning in older adults with mild cognitive impairment: a systematic review and meta-analysis of randomized controlled trials
Source: Front Public Health. 2026 Jan 16;13:1682781. doi: 10.3389/fpubh.2025.1682781 (PMC12855459; doi:10.3389/fpubh.2025.1682781)
Supplement: Supplementary file 1 [file Supplementary_file_1.pdf]

Appendix Table 1. Baseline Cognitive Function: Supplementary Data

21 studies (including high risk studies)					11 studies (excluding high risk studies)					11 studies (excluding high risk studies) — including single study meta analyses				
Subgroup	SMD_CI	P_value	I^2	Tau2	Subgroup	SMD_CI	P_value	I^2	Tau2	Subgroup	SMD_CI	P_value	I^2	Tau2
Overall (n=21)	0.50 [0.31, 0.68]	<0.001	52.60%	0.091	Overall (n=21)	0.69 [0.43, 0.95]	<0.001	52.80%	0.101	Overall (n=11)	0.69 [0.43, 0.95]	<0.001	52.80%	0.101
Function-based VR					Function-based VR					Function-based VR				
Purely Cognitive VR (n=8)	0.63 [0.25, 1.01]	0.001	72.60%	0.213	Purely Cognitive VR (n=4)	0.97 [0.43, 1.51]	<0.001	77.30%	0.231	Purely Cognitive VR (n=4)	0.97 [0.43, 1.51]	<0.001	77.30%	0.231
Exergaming VR (n=9)	0.47 [0.26, 0.68]	<0.001	2.90%	0	Exergaming VR (n=6)	0.53 [0.29, 0.78]	<0.001	0.00%	0	Exergaming VR (n=6)	0.53 [0.29, 0.78]	<0.001	0.00%	0
Cognitive-motor VR (n=4)	0.28 [0.08, 0.48]	0.006	9.20%	0						Cognitive-motor VR (n=1)	0.37 [-0.31, 1.05]	0.285	0.00%	0
Immersion-based VR					Immersion-based VR					Immersion-based VR				
Non-Immersive VR (n=4)	0.38 [0.13, 0.63]	0.003	0.00%	0	Non-Immersive VR (n=2)	0.48 [0.14, 0.83]	0.006	0.00%	0	Non-Immersive VR (n=2)	0.48 [0.14, 0.83]	0.006	0.00%	0
Immersive VR (n=13)	0.57 [0.31, 0.82]	<0.001	65.30%	0.134	Immersive VR (n=7)	0.78 [0.41, 1.16]	<0.001	66.40%	0.166	Immersive VR (n=7)	0.78 [0.41, 1.16]	<0.001	66.40%	0.166
Semi-Immersive VR (n=4)	0.29 [-0.29, 0.87]	0.322	46.50%	0.16	Semi-Immersive VR (n=2)	0.57 [0.01, 1.12]	0.045	0.00%	0	Semi-Immersive VR (n=2)	0.57 [0.01, 1.12]	0.045	0.00%	0
Control group type					Control group type					Control group type				
Passive control (n=7)	0.44 [-0.04, 0.92]	0.073	75.60%	0.299	Passive control (n=4)	0.84 [0.30, 1.37]	0.002	61.20%	0.177	Passive control (n=4)	0.84 [0.30, 1.37]	0.002	61.20%	0.177
Active control (n=14)	0.50 [0.32, 0.69]	<0.001	23.50%	0.032	Active control (n=7)	0.61 [0.33, 0.90]	<0.001	45.00%	0.068	Active control (n=7)	0.61 [0.33, 0.90]	<0.001	45.00%	0.068
Duration (weeks)					Duration (weeks)					Duration (weeks)				
≤6 (n=7)	0.39 [0.14, 0.64]	0.002	23.50%	0	≤6 (n=3)	0.48 [0.12, 0.84]	0.009	0.00%	0	≤6 (n=3)	0.48 [0.12, 0.84]	0.009	0.00%	0
8–12 (n=10)	0.59 [0.26, 0.92]	<0.001	70.80%	0.181	8–12 (n=6)	0.83 [0.39, 1.28]	<0.001	69.10%	0.203	8–12 (n=6)	0.83 [0.39, 1.28]	<0.001	69.10%	0.203
>12 (n=4)	0.46 [0.19, 0.74]	<0.001	6.60%	0.015	>12 (n=2)	0.59 [0.22, 0.96]	0.002	23.20%	0.017	>12 (n=2)	0.59 [0.22, 0.96]	0.002	23.20%	0.017
Frequency (times)					Frequency (times)					Frequency (times)				
≤2 (n=14)	0.50 [0.22, 0.78]	<0.001	67.00%	0.18	≤2 (n=7)	0.84 [0.48, 1.20]	<0.001	56.70%	0.129	≤2 (n=7)	0.84 [0.48, 1.20]	<0.001	56.70%	0.129

≥3 (n=7)	0.46 [0.25, 0.66]	<0.001	0.00%	0	≥3 (n=4)	0.43 [0.17, 0.70]	0.001	0.00%	0	≥3 (n=4)	0.43 [0.17, 0.70]	0.001	0.00%	0
Session (minutes)					Session (minutes)					Session (minutes)				
41–60 (n=10)	0.58 [0.23, 0.92]	0.001	74.40%	0.221	41–60 (n=7)	0.84 [0.47, 1.20]	<0.001	57.00%	0.133	41–60 (n=7)	0.84 [0.47, 1.20]	<0.001	57.00%	0.133
>60 (n=4)	0.54 [0.23, 0.86]	<0.001	0.00%	0.006	>60 (n=2)	0.55 [0.07, 1.04]	0.026	49.20%	0.061	>60 (n=2)	0.55 [0.07, 1.04]	0.026	49.20%	0.061
≤40 (n=7)	0.37 [0.15, 0.59]	0.001	0.00%	0	≤40 (n=2)	0.39 [0.01, 0.77]	0.043	0.00%	0	≤40 (n=2)	0.39 [0.01, 0.77]	0.043	0.00%	0

Appendix Figure 1. Subgroup Analysis Stratified by Cognitive Measurement Tools

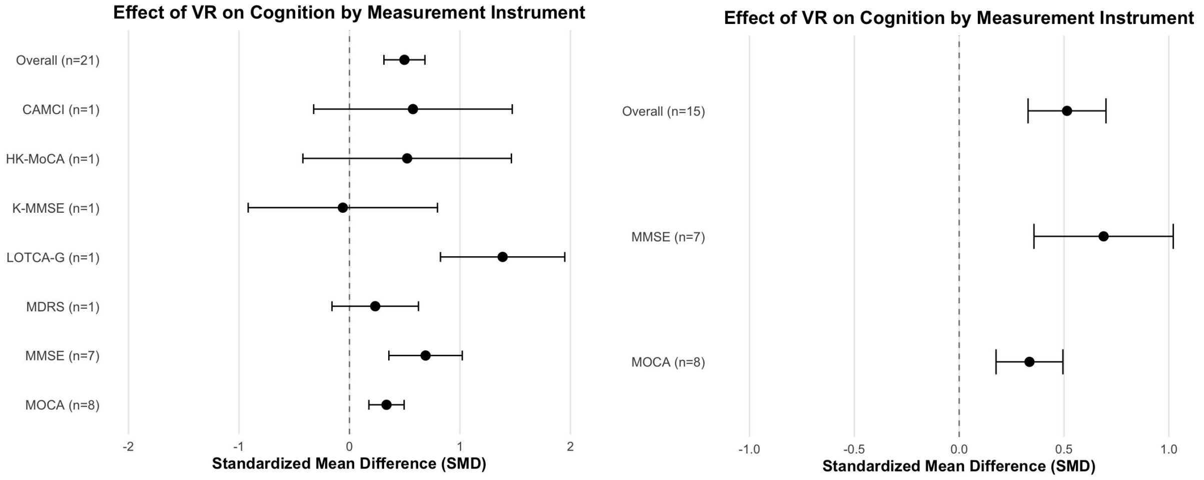

Appendix Explanatory Note for Appendix

The protocol was pre-registered with the International Prospective Register of Systematic Reviews, under registration number CR D420251002107 (https://www.crd.york.ac.uk/PROSPERO/view/CRD420251002107).
